# Renin Inhibition with Aliskiren: A Decade of Clinical Experience

**DOI:** 10.3390/jcm6060061

**Published:** 2017-06-09

**Authors:** Nikolaos-Dimitrios Pantzaris, Evangelos Karanikolas, Konstantinos Tsiotsios, Dimitrios Velissaris

**Affiliations:** 1 Medical School, University of Patras, Rio Achaia 26504, Greece; npantzaris@gmail.com; 2 Department of Medicine, Schools of Health Sciences, University of Athens, 75 Mikras Asias Str., Athens 11527, Greece; evangelos.karanikolas@gmail.com; 3 Internal Medicine Department, University Hospital of Patras, Rio Achaia 26504, Greece; konstantinos.tsiotsios@gmail.com

**Keywords:** aliskiren, hypertension, direct renin inhibitors, renin-angiotensin-aldosterone system

## Abstract

The renin-angiotensin-aldosterone system (RAAS) plays a key role in the pathophysiology of arterial hypertension as well as in more complex mechanisms of cardiovascular and renal diseases. RAAS-blocking agents like angiotensin-converting enzyme (ACE) inhibitors and angiotensin II receptor blockers, have long been key components in the treatment of essential hypertension, heart failure, diabetic nephropathy, and chronic kidney disease, showing benefits well beyond blood pressure reduction. Renin blockade as the first step of the RAAS cascade finally became possible in 2007 with the approval of aliskiren, the first orally active direct renin inhibitor available for clinical use and the newest antihypertensive agent on the market. In the last decade, many clinical trials and meta-analyses have been conducted concerning the efficacy and safety of aliskiren in comparison to other antihypertensive agents, as well as the efficacy and potential clinical use of various combinations. Large trials with cardiovascular and renal endpoints attempted to show potential benefits of aliskiren beyond blood pressure lowering, as well as morbidity and mortality outcomes in specific populations such as diabetics, heart failure patients, and post-myocardial infarction individuals. The purpose of this review is to present the currently available data regarding established and future potential clinical uses of aliskiren.

## 1. Introduction

Elevated blood pressure, defined as SBP > 140 mm Hg or DBP > 90 mm Hg or pharmacologically achieved normal BP, has an estimated prevalence of 40% in adults over 25 years old [1] and is among the leading risk factors for disease burden [2]. As it is a well-established risk factor for CVD, the leading cause of mortality around the globe [3], prompt treatment is essential both for the patient and the health care system. Recent data also suggest that intensive elevated BP treatment to lower goals (<120 mm Hg) may have a beneficial role by reducing cardiovascular events and all-cause mortality [4].

**Figure 1:**
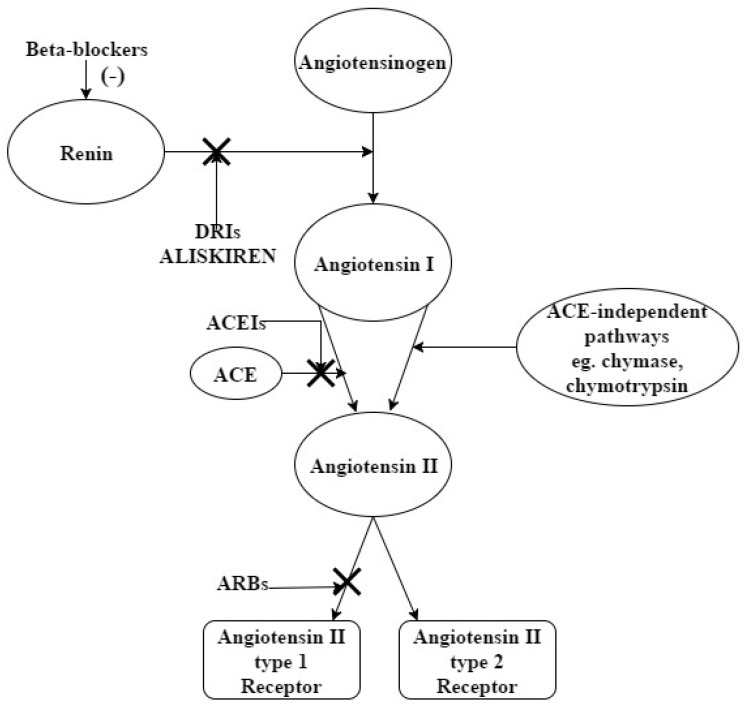
RAAS and its pharmacological blockade.

The renin angiotensin aldosterone system (RAAS) (Figure 1) plays a pivotal role in BP regulation, thus drugs targeting steps in the cascade—such as ACEIs and ARBs—are widely used as antihypertensive agents. Renin is the first and highly regulated rate-limiting step of the system, and its inhibition has been a target for nearly 60 years. Recently, a new receptor was discovered, able to bind renin and prorenin and increase the conversion of angiotensinogen to Ang I [5], but its role in hypertension and pharmacological renin inhibition remains to be clarified [6]. Although ACEIs and ARBs block Ang II biologic actions, they increase plasma renin activity (PRA), the rate of conversion of angiotensinogen to Ang I by renin, by interrupting the negative feedback loop of renin release. Elevated PRA levels seem to be predictive of higher mortality and major cardiovascular events [7,8], but a recent large retrospective cohort study supported those findings only among individuals with SBP ≥ 140 mm Hg [9]. Also, the reactive rise in Ang I levels by the ACEIs leads to Ang II formation in the tissues by ACE-independent pathways, like chymase and chymotrypsin, and consequently loss of their BP-lowering efficacy, a phenomenon known as ACE-escape [10].

Aliskiren (ALI) is the first non-peptide, orally active, highly potent, and selective inhibitor of human renin [10,11,12] approved for use in the treatment of hypertension. ALI has a 2.5% oral bioavailability, a long half-life of approximately 40 h, and is mainly excreted through bile via the fecal route [13]. Significant drug interactions have not yet been identified [14], and dosing adjustment is not required in patients with liver disease [15] or in patients with renal impairment [16]. The pharmacokinetics of ALI show a high inter-subject variability [17]. ALI shows a dose-dependent decrease in Ang II levels [18], and BP-lowering effects in doses between 75–600 mg. The small efficacy of the 75 mg dose and higher incidence of adverse events (AEs) with no additional benefit of the 600 mg dose [19] resulted in the clinical use of 150 and 300 mg dosing regimens. Although ALI shows a great reactive rise in plasma renin concentration (PRC)—higher than the elevation caused by ACEIs and ARBs—it greatly suppresses PRA, unlike the other RAAS-blocking agents, and this elevation in PRC is not associated with paradoxical increases in BP in patients with hypertension [20]. Also, renin inhibition with ALI reaches above 99% in the first hours following administration and remains above 95% 24 h later. The reactive rise in PRC caused by ALI is much lower than the 20- to 100- fold rise required to overcome those percentages of inhibition [21].

The studies published up to February 2017, regarding clinical trials for the use of ALI in the treatment of essential hypertension as a monotherapy or as part of a combined treatment are presented along with their main findings in Table 1.

## 2. Aliskiren and Blood Pressure Lowering

### 2.1. Monotherapy

Several randomized control trials conducted showed significant dose-related BP lowering effects with ALI monotherapy [18,21,22,23,24,25,26,27,28,29,30], similar to those observed with losartan (LOS) [22], valsartan (VAL) [25], irbesartan IRB) [47], and lisinopril (LIS) [28], and a placebo-like tolerability profile. A meta-analysis of six double-blind RCTs comparing ALI to placebo supported those findings [82]. Some studies concluded that ALI has superior BP-lowering effects, and higher control rates than ramipril (RAM) [28,30,33,34] but others did not [78]. A pooled analysis of three clinical trials by Verdecchia et al. showed that overall SBP was lower with ALI than with RAM, results were attributed to the ACE-escape phenomenon of RAM-based treatment and the longer half-life of ALI versus RAM (40 h vs. 15 h) [83]. In 2011, two meta-analyses published concluded that ALI is equally effective with ARBs with a similar AE profile [84,85]. Another meta-analysis conducted in 2013 by Chen et al., including 14 studies with a total of 6741 participants, found that ALI is as effective as ARBs although it had higher control rates. ALI was also proven superior to ACEIs in DBP reductions, similar to hydrochlorothiazide (HCTZ), and inferior to CCBs in BP reduction and control rates [86].

ALI also shows more sustained BP-lowering effects than telmisartan (TEL) after treatment withdrawal [64] and RAM and IRB after a missed dose simulation [47]. This feature of ALI, partially attributed to the long drug half-life, is of great importance, as patient compliance is a crucial issue in patients trying to achieve BP control and missed doses are quite a common phenomenon in everyday clinical practice.

### 2.2. Combination

As combination treatment is quite often required by patients to achieve optimal BP targets, many studies conducted tested combinations of ALI with HCTZ, ARBs, CCBs, and beta-blockers. ALI combined with HCTZ produced greater BP reductions and higher control rates than either drug alone [41,42,43,44] with similar tolerability and a higher incidence of hypokalemia in HCTZ monotherapy. ALI also neutralizes the reactive PRA increase caused by HCTZ [26].

Studies of ALI/amlodipine (AML) combinations showed that doses of 300–150/10 mg are more effective than AML 10 mg monotherapy and have a significantly lower incidence of peripheral edema [46,47]. Two similar trials testing ALI/HCTZ 300/25 mg and AML 10 mg had slightly differing results. One of them including patients with DM, concluded that the combination produced greater msSBP reductions [53] and the other found both treatments similar in both msSBP/msDBP reductions [56]. These results might indicate a different efficacy profile of the dual combination in patients with DM. In the ACCELERATE study, Brown et al. concluded that ALI/AML 300/10 mg causes higher SBP reductions than either agent alone and is recommended as the initial therapy if the patient’s SBP is greater than 150 mm Hg [54]. In a meta-analysis of seven randomized control trials by Liu et al., ALI/AML combination was found to be more effective than either component monotherapy [87].

Liu et al., in 2014 published another meta-analysis including 19 trials and 13,614 participants comparing ALI/HCTZ and ALI/AML. The data showed that combination therapies were more efficient than the respective monotherapies and that ALI/AML produced significantly greater SBP/DBP reductions, and higher response and control rates [88]. Triple combinations with ALI/AML/HTCZ 300/10/25 mg have also shown similar tolerability and higher efficacy with significantly larger msSBP/msDBP reductions and higher control rates as compared to the components’ dual combinations in patients with moderate-to-severe hypertension [53,54,55].

Trials testing combinations of ALI with ARBs showed additive BP lowering effects and similar tolerability profiles to each agent monotherapy, and a low rate of potassium elevations [24,56,57,58]. Also, the reactive PRA rise by the ARB therapy is blunted by the ALI co-administration. Although these studies, as well as a large meta-analysis [85], show the greater BP-lowering potential of ALI/ARB combinations, dual RAAS blockade does not seem to reduce overall and cardiovascular mortality and it is associated with a higher risk of AEs (hypotension, hyperkalemia, renal failure) [89].

In the only randomized control trial including a beta-blocker with 694 participants in 2008, Dietz et al. concluded that ALI/atenolol (ATEN) produced greater reductions than either monotherapy and msDBP reductions with ATEN were significantly higher than with ALI alone. ALI treatment was not associated with bradycardia and had fewer AEs and discontinuations. All three regimens reduced mean PRA (as also expected with beta-blocker monotherapy caused by reduced renin secretion from the juxtaglomerular cells due to beta blockade) [35].

### 2.3. Special Populations

In the first large trial conducted in 837 patients with DM, Uresin et al. concluded that ALI/RAM 300/10 mg produced significantly greater msDBP reductions than either agent alone. ALI monotherapy was superior to RAM monotherapy for msSBP reduction and non-inferior for msDBP reduction [32]. The addition of ALI 300 mg in the VAL/HCTZ 160/25 mg regimen in diabetic individuals showed greater BP reductions, however this did not achieve statistical significance [60]. In a recent randomized control trial by Imbalzano et al., ALI addition to optimal antihypertensive therapy showed higher BP and microalbuminuria reductions than LOS or RAM addition in patients with uncontrolled BP and DM [79].

Obese individuals also constitute a unique and challenging group of patients, as they have a higher prevalence of hypertension and very low adequate blood pressure control rates [90]. Jordan et al. in a study including 489 participants with a BMI of 30 or greater that had previously failed to reach BP targets with HCTZ monotherapy, found that ALI/HCTZ combination was superior to placebo/HCTZ and similar to AML/HCTZ and IRB/HCTZ in BP-lowering, with a tolerability similar to placebo/HCTZ [27]. In a subgroup analysis of 396 obese patients with hypertension, although ALI-based therapy demonstrated similar mean BP reductions in obese and non-obese individuals, HCTZ-based therapy showed significantly lower mean BP reductions in obese than in non-obese patients, suggesting that ALI is a superior treatment to HCTZ in obese patients with hypertension [91].

When ALI was compared to IRB in patients with metabolic syndrome and hypertension, ALI showed significantly greater mean BP reductions and almost double target BP control rates, with both treatments showing similar effects on glucose and lipid profiles. Also, both treatments showed small, non-statistically significant changes in a panel of inflammatory and cardiovascular risk biomarkers [59]. In another trial with 76 individuals with metabolic syndrome, ALI and LOS produced similar SBP/DBP reductions, but ALI improved insulin sensitivity and fibrinolytic balance by not changing tPA activity, that decreased with LOS treatment [48].

Blood pressure control in the elderly is also a field of great challenges. In hypertensive individuals aged 65 years or older, ALI in doses of 75, 150, and 300 mg showed significant BP-lowering effects compared to placebo with an estimated minimum effective dose of 81.9 mg [68]. In two randomized, controlled trials with participants over 65 years old, ALI demonstrated no difference compared to LIS in BP efficacy [29] and was found superior to RAM monotherapy in BP reductions as well as in control rates [43]. In the most recent trial in an elderly Japanese population, ALI/AML 150–300/5 mg and AML 10 mg monotherapy showed similar BP-lowering profiles but ALI/AML combination was significantly less efficient in reducing the early morning BP and the morning BP surge compared to high dose AML [80].

### 2.4. Real Life Data

Although numerous clinical trials and meta-analyses have been conducted to date, the fact that ALI is the newest antihypertensive agent available on the market, raises the need for efficacy and safety data, with uncontrolled conditions in the setting of everyday clinical practice. In the Belgian prospective observational DRIVER study, 1695 patients whose prior treatment was inadequate or not tolerated, completed a 180-day treatment regimen with ALI. At the end of treatment, mean SBP/SDP reductions were 22.9 ± 16.7/10.5 ± 10.9 mm Hg (*p* < 0.001). Adequate BP control based on 2009 guidelines was achieved by 56.3% of patients (*p* < 0.001) and 64.2% of eligible patients had a CV risk reduction [92]. In data derived from the Italian web-based drug-monitoring system, ALI prescribed in patients with uncontrolled BP and organ damage or comorbidities produced lower SBP/DBP measurements consistently on follow-up visits, and very few reported AEs [93].

A large observational, multicenter, multiethnic study from 420 centers in Asia and the Middle East included 4826 patients with hypertension receiving ALI or ALI/HCTZ treatment. Both ALI and ALI/HCTZ showed significant msSBP/DBP reductions, 24.1/12.2 mm Hg and 27.6/14.1 mm Hg respectively, and very high response rates [94]. The 3A registry, a prospective cohort study of 13,433 patients from Germany, compared the efficacy in real practice of ALI or ACEI/ARB or a non-RAAS blocking agent alone, or as an addition to an existing regimen. One year outcomes showed no significant differences in BP reduction between the three groups after confounders and baseline BP adjustments. The mean number of antihypertensive agents used was higher in the ALI group but ALI was most often prescribed in patients with higher BP baseline and concomitant diseases (chronic heart failure, diabetes, ischemic heart disease, and renal disease) [95].

Recently in 2015, RALLY, a three-month observational study with 566 hypertensive patients treated and followed by 140 physicians, showed the efficacy and tolerability of ALI/AML combination. SBP and DBP were on average reduced from 161 ± 14 to 135 ± 10 mm Hg and 93 ± 9 to 81 ± 6 mm Hg, respectively with 94% of the patients being compliant to therapy [96].

## 3. Aliskiren and End-Organ Damage

Over the last decade, large-scale, long-term trials have been designed and conducted investigating the possible role of ALI in the prevention of end-organ damage and on morbidity and mortality outcomes beyond its blood pressure lowering effects in specific high-risk populations. Though early data seemed very promising, more recent data published raised many questions and new concerns to be addressed in further trials in the future. To date, 10 concluded trials have published their results, with the latest published in early 2016. A summary of these trials along with their main findings are presented in Table 2.

### 3.1. Diabetics

In the AVOID study, Parving et al. investigated the possible renoprotective effects of dual RAAS blockade by adding ALI in the maximum recommended dose of LOS 100 mg in a multinational, randomized, controlled, double-blind study, enrolling 599 patients with DM and nephropathy. Treatment with ALI significantly reduced Urine Albumin-to-Creatinine Ratio (UACR) compared to placebo, and both groups had a similar incidence of AEs and serious AEs. BP reductions were similar in both groups. Thus ALI’s renoprotective effect was suggested to be independent of its BP-lowering effect [97].

Despite the early promising results from the AVOID trial, the ALTITUDE trial that enrolled 8561 patients with DM and CKD, CVD, or both to test the effects of ALI added to an ACEI or an ARB, had to be prematurely terminated on the basis of futility and safety reasons. An increased number of AEs (renal dysfunction, hyperkalemia, and hypotension) with no added benefit and a higher incidence of non-fatal strokes in the ALI group compared to placebo were the main concerns [107]. In the published results, the authors concluded that the addition of ALI in standard ACEI or ARB treatment is contraindicated in patients with DM and cardiovascular or renal disease [102].

In a study conducted by Bakris et al., before the ALTITUDE trial discontinuation, in 1143 hypertensive individuals with DM and stage 1 or 2 CKD, the ALI/VAL 150/160 mg combination was found to have additive BP-lowering effects and similar tolerability to VAL 160 mg monotherapy. The authors attributed those different safety findings to the level of kidney function at baseline and the study duration. Bakris et al. did not include patients with eGFR <60 mL/min/1.73 m^2^ or CVD in their trial though, which was the patient profile in ALTITUDE [76].

### 3.2. Left Ventricular Hypertrophy

The ALLAY trial enrolled 465 patients with hypertension, BMI > 25 kg/m^2^ and increased ventricular wall thickness. Patients received ALI 300 mg, or LOS 100 mg, or a combination. After a nine-month period, left ventricular mass index was significantly reduced in all treatment groups. ALI was non-inferior to LOS in reducing left ventricular hypertrophy, and the reduction in the combination arm was not significantly different from that in the LOS monotherapy arm [99].

### 3.3. Acute Coronary Syndromes

The effect of ALI, VAL, and their combination was studied in 1101 stable patients after an acute coronary syndrome with no evident HF or left ventricular function ≤40% but with elevated natriuretic peptides 3–10 days after admission in the AVANT GARDE-TIMI 43 trial. The reduction of NT-proBNP levels from baseline to week 8 (primary endpoint) was similar in all groups; 44% in aliskiren, 39% in valsartan, 36% in the combination arm, and 42% in placebo. Patients receiving active therapy had a higher incidence of AEs and serious AEs with no differences in clinical outcomes [100].

The addition of ALI to standard optimal therapy (ACEI or ARB and beta-blocker) compared to the addition of placebo was compared in the ASPIRE study in 820 post-MI patients with the LVEF ≤ 45%, and regional wall motion abnormalities. The addition of ALI did not produce any change in left ventricular end-systolic volume compared to placebo. The incidence of cardiovascular death and hospitalization for HF were also similar in both groups. The serious AEs were similar in both arms, but the ALI arm had a larger number of AEs (hyperkalemia, hypotension, and creatinine elevation) [101].

### 3.4. Elderly

The APOLLO trial aimed to follow and test the effects on CVD of ALI 300 mg vs. placebo with the optional addition of HCTZ or AML vs. placebo in 11,000 elderly individuals with SBP ≥ 130 mm Hg and <160 mm Hg for a five-year period. Due to early termination by the sponsor, a total of 1759 individuals were finally randomized with a median follow-up time of 0.6 years. After the recruitment discontinuation, given the recent results from ALTITUDE, instructions were given to stop ALI or placebo in diabetic patients receiving an ACEI or an ARB. The original study objectives regarding clinical outcomes could not be reached. The data suggested a potential benefit in clinical outcomes with the use of multiple BP-lowering agents in elders with stage 1 hypertension [104].

### 3.5. Coronary Atherosclerosis

The comparison of ALI vs. placebo in 613 patients with coronary artery disease, prehypertension (125 mm Hg ≤ SBP < 140 mm Hg), and two additional cardiovascular risk factors, was the objective of the AQUARIUS trial. Both primary and secondary efficacy parameters, percent atheroma volume, and normalized total atheroma volume respectively, did not significantly differ between the two groups. The proportion of patients demonstrating regression of percent atheroma volume was also similar in both groups. Thus no benefit was shown by the use of ALI in prehypertensive individuals with coronary atherosclerosis [103].

### 3.6. Heart Failure

In the ALOFT trial, including 302 patients with HF receiving an ACEI and a beta-blocker, the addition of ALI showed significant plasma NT-proBNP reductions compared to placebo with no important BP differences between the two groups. Urinary aldosterone was also reduced in the ALI arm [98]. Those results were different from those reported by the AVANT GARDE-TIMI 43 trial described above, but the significantly different study population (No evident HF in AVANT GARDE-TIMI 43) must be taken into account.

The two more recent large randomized, controlled trials regarding ALI in HF patients showed disappointing results. In the ASTRONAUT study enrolling 1639 participants with a LVEF ≤ 40%, elevated natriuretic peptides and symptoms of fluid overload, the addition of ALI to standard treatment did not reduce cardiovascular death or HF rehospitalization compared to placebo and showed a higher incidence of AEs (hyperkalemia, hypotension, and renal dysfunction) [105]. The long-awaited results from the ATMOSPHERE trial were also discouraging regarding the use of ALI in HF patients. After an approximately four-year mean follow-up time, 7016 participants were randomized into three groups, ALI, enalapril (ENA), or both, and the primary outcome was death from a cardiovascular cause or hospitalization for HF. ALI non-inferiority was not proven compared to ENA, and their combination had an increased risk of hypotension, hyperkalemia, and serum creatinine level elevations without any additional benefit [106].

## 4. Conclusions

Many of the recent findings suggest that there may be an upper limit in RAAS blockade, in terms of benefit versus safety and tolerability, especially in specific higher risk populations. ALI is now a well-established antihypertensive agent, but its optimal use remains to be further tested. Though specific populations seem to benefit more from direct renin inhibition by ALI (e.g., obese, metabolic syndrome and resistant hypertension) for others, it is just another viable option in the armory of clinicians to achieve adequate BP control. As many patients often require multidrug antihypertensive therapy, ALI, the only available direct renin inhibitor in the market, can play an important role as a RAAS-blocking drug option in combination regimens. Yet ALI treatment still has a higher financial cost, when compared to other RAAS-inhibitors and non RAAS-blocking drugs.

The possible benefits of ALI on other physiological targets, such as endothelial function and arterial stiffness, on which recent studies have suggested favorable effects [108,109,110] warrant further investigation. Also, the exact clinical implications in the role of disease of the (pro)renin receptor and PRA levels should be established, as ALI has an effect on both. Despite the recent discouraging results on several morbidity and mortality endpoints in large prospective trials, there is a need for longitudinal studies assessing ALI alone and in combination to identify the specific subgroups of patients that would benefit more from direct renin blockade and the biomarkers needed to monitor those effects.

## Figures and Tables

**Table 1:** Summary of studies.

First Author	Year/Country Study Type	Arms	Participants	Main Findings
Stanton A. [22]	2003 Ireland RCT	ALI 37.5; 75; 150; 300; LOS 100	226 Mild-to-moderate hypertension	Dose-dependent reductions in ambulatory BP and decreased PRA were observed with ALI. Changes following 75, 150, and 300 mg of ALI were not significantly different to those observed with 100 mg of LOS.
Gradman A.H. [23]	2005 USA RCT	ALI 150; 300; 600; IRB 150; Placebo	652 Mild-to-moderate hypertension	Oral ALI showed an effective BP lowering effect. ALI 300 and 600 mg lowered msDBP significantly more than IRB 150 mg. ALI 150 mg had a similar antihypertensive effect to IRB 150 mg.
Kushiro T. [24]	2006 Japan RCT	ALI 75; 150; 300; Placebo	455 Japanese Mild-to-moderate hypertension	Once-daily oral ALI provided significant, dose-dependent reductions in msSBP/DBP with placebo-like tolerability in Japanese patients with hypertension.
Pool J.L. [25]	2007 Multicenter RCT	ALI 75; 150; 300; VAL 80; 120; 360; ALI/VAL; VAL/HCTZ; Placebo	1123 Mild-to-moderate hypertension	ALI monotherapy produced dose-related reductions in DBP/SBP similar to VAL monotherapy and placebo-like tolerability. The combination of the two showed additive results with similar tolerability.
O’Brien E. [26]	2007 Ireland RCT	ALI 150; RAM 5; IRB 150; ALI/HCTZ; ALI/RAM; ALI/IRB	67 Mild-to-moderate hypertension	ALI combined with HCTZ, RAM, or IRB, demonstrated significantly greater BP reductions than any agent monotherapy and also neutralized the compensatory rise in PRA stimulated by the other antihypertensive agents.
Jordan J. [27]	2007 Multicenter RCT	ALI/HCTZ; AML/HCTZ; IRB/HCTZ; Placebo/HCTZ	489 Obese Mild-to-moderate hypertension	ALI/HCTZ showed significantly greater BP reductions than HCTZ alone, but similar to AML/HCTZ and IRB/HCTZ in obese hypertensive individuals. The ALI combination also neutralized the compensatory rise in PRA.
Strasser R.H. [28]	2007 Multicenter RCT	ALI 300; LIS 40; ALI/HCTZ; LIS/HCTZ	153 Severe hypertension	ALI 300 mg monotherapy or ALI/HCTZ combination demonstrated similar efficacy and tolerability to LIS 40 mg monotherapy or LIS/HCTZ combination respectively in patients with severe hypertension.
Verdecchia P. [29]	2007 Multicenter RCT	ALI 75; 150; 300; LIS 10	355 (≥65 years old) Mild-to-moderate hypertension	ALI 75/150/300 mg and LIS 10mg lowered ambulatory SBP and office msSBP with no significant difference between ALI doses and no evidence of dose-related increases in the incidence of AEs.
Oh B.H. [19]	2007 Multicenter RCT	ALI 150; 300; 600; Placebo	672 Mild-to-moderate hypertension	ALI 150 to 600 mg once daily, provided significant antihypertensive efficacy and placebo-like tolerability. The lowering effect of ALI 600 mg was greater but not statistically significant of that of ALI 300 mg.
Drummond W. [30]	2007 Multicenter RCT	AML 5; 10; ALI/AML	545 Mild-to-moderate hypertension	ALI/AML 150/5 mg demonstrated greater BP lowering efficacy than AML 5 mg monotherapy but similar to AML 10 mg, although less edema was noted with the combination treatment.
Oparil S. [31]	2007 Multicenter RCT	ALI 300; VAL 320; ALI/VAL; Placebo	1797 Mild-to-moderate hypertension	ALI/VAL 300/320 mg significantly lowered msDBP as compared to either component monotherapy or placebo. Safety and tolerability profiles were similar in all groups.
Uresin Y. [32]	2007 Multicenter RCT	ALI 300; RAM 10; ALI/RAM	837 DM Mild-to-moderate hypertension	ALI 300 mg provided additional, significant BP reductions when combined with RAM 10 mg in patients with hypertension and diabetes mellitus.
Villamil A. [33]	2007 Multicenter RCT	ALI 75; 150; 300; HCTZ 6.25; 12.5; 25; ALI/HTCZ; Placebo	2776 Mild-to-moderate hypertension	ALI/HCTZ combination produced a greater reduction in BP measurements and achieved better control rates and more responders than either monotherapy. HCTZ monotherapy increased PRA, but PRA decreased in the ALI monotherapy and the combination groups
Andersen K. [34]	2008 Iceland RCT	ALI 300; RAM 10; ALI/HCTZ; RAM/HCTZ	687 Mild-to-moderate hypertension	ALI based therapy, alone or with HCTZ, produced greater msSBP/DBP reductions, better control rates, and more sustained effects after drug discontinuation than RAM based therapy.
Dietz R. [35]	2008 Multicenter RCT	ALI 300; ATEN 100; ALI/ATEN	694 Mild-to-moderate hypertension	ALI/ATEN induced significantly greater msSBP reductions than ALI or ATEN alone, and msDBP reductions were larger with ATEN than with ALI. All three regimens reduced mean PRA from baseline. ALI treatment had lower rates of AEs and was not associated with bradycardia.
Nickenig G. [36]	2008 Multicenter RCT	ALI 300; ALI/HCTZ	880 Non-responders to ALI monotherapy Mild-to-moderate hypertension	ALI/HCTZ 300/25 mg and 300/12.5 mg produced significantly greater msSBP/DBP reductions from baseline as well as higher BP control rates than ALI 300 mg alone with similar tolerability.
Geiger H. [37]	2009 Multicenter RCT	HCTZ 25; ALI/HCTZ; VAL/HCTZ; ALI/VAL/HCTZ	641 Non-responders to HCTZ monotherapy; Mild-to-moderate hypertension	The ALI/VAL/HCTZ 300/320/25 mg arm produced statistically significant additional reductions in SBP/DBP and higher control rates than the ALI/HCTZ 300/25 mg, VAL/HCTZ 320/25 mg combinations and the HCTZ 25 mg monotherapy. The safety profile of the triple combination was similar to that of the double combinations.
Kushiro T. [38]	2009 Multicenter RCT	ALI 300; ALI/diuretic; ALI/CCB	345 Japanese Mild-to-moderate hypertension	ALI monotherapy, as well as combinations of ALI/diuretic and ALI/CCB, achieved clinically significant msSBP/DBP reductions. All regimens were well tolerated, and the overall responder rate was high.
Puig J.G. [39]	2009 Multicenter RCT	ALI 75; 150; 300; Placebo	642 Mild-to-moderate hypertension	Dose-related BP reductions were observed with ALI 75, 150, and 300 mg, but only the reductions achieved with ALI 150 and 300 mg were statistically significant compared to placebo. All doses were well tolerated.
Blumenstein M. [40]	2009 Multicenter RCT	HCTZ 25; ALI/HCTZ 150/25; 300/25	722 Non-responders to HCTZ monotherapy; Mild-to-moderate hypertension	Single pill ALI/HCTZ 300/25 mg and 150/25 mg combinations significantly lowered msSBP/DBP with the 300/25 mg combination producing the greater reductions. Responder rates were also significantly higher with ALI/HCTZ combinations. ALI/HCTZ showed similar tolerability to HCTZ monotherapy and a numerically lower incidence of hypokalemia.
Littlejohn T.W. III [41]	2009 Multicenter Non RCT	ALI/AML 300/10 ± HCTZ	556 Mild-to-moderate hypertension	ALI/AML 300/10 mg, with or without add-on HCTZ, effectively reduced BP, especially in patients with stage 2 hypertension. The most common AE was peripheral edema.
Schmieder R.E. [42]	2009 Multicenter RCT	ALI 300; HCTZ 25; Placebo ± AML	1124 Mild-to-moderate hypertension	ALI based therapy produced significantly greater BP reductions and higher responder rates than HCTZ based therapy. AE rates were similar in all groups with hypokalemia being more frequent in HCTZ based therapy.
Duprez D.A. [43]	2010 USA RCT	ALI 300; RAM 10 ± HCTZ or AML	901 (≥65 years old) Mild-to-moderate hypertension	ALI monotherapy in elders with essential hypertension showed greater msSDB/DBP reductions and higher control rates than RAM monotherapy. Also fewer patients required add-on treatment with HCTZ or AML. Tolerability was similar, but the RAM group had a higher incidence of cough.
Black H.R. [44]	2010 Multicenter RCT	ALI 300; ALI/HCTZ	688 160 mm Hg ≤ SBP < 180 mm Hg	ALI 300 mg monotherapy, as well as ALI/HCTZ 300/25 mg, produced substantial BP reductions from baseline with the reductions being significantly greater in the combination therapy.
Ito S. [45]	2010 Japan Non RCT	ALI 75→300	40 Japanese Renal dysfunction; Mild-to-moderate hypertension	65% of the patients achieved BP response and 30% BP control with ALI monotherapy. The AE profile was low, and similar to studies with hypertensive patients without renal dysfunction.
Chrysant S.G. [46]	2010 Multicenter Non RCT	ALI/VAL 300/320 ± HCTZ	601 Mild-to-moderate hypertension	ALI/VAL 300/320 mg with or without optional HCTZ addition showed clinically significant BP-lowering effects, high BP control rates and was well-tolerated with a very low incidence of hyperkalemia in patients with hypertension.
Palatini P. [47]	2010 Multicenter RCT	ALI 300; IRB 300; RAM 10	654 Mean ambulatory DBP ≥ 85 mm Hg	ALI 300 mg provided similar BP-lowering effects with IRB 300 mg and significantly greater than RAM 10 mg. The maintenance of the mean ambulatory SBP/DBP lowering effect of ALI was significantly greater when compared to both IRB and RAM monotherapies.
Fogari R. [48]	2010 Italy RCT	ALI 300; LOS 100	76 Metabolic syndrome Mild-to-moderate hypertension	Both ALI 300 mg and LOS 100 mg induced a significant and similar SBP/DBP reduction. tPA activity decreased with LOS and did not change significantly with ALI. Insulin sensitivity was also improved with ALI and remained unchanged with LOS.
Weir M.R. [49]	2010 Multicenter RCT	ALI 300 + ≥ 200 mmol/d Na; ALI 300 + ≤ 100 mmol/d Na	132 135 mm Hg ≤ SBP < 160 mm Hg	During ALI 300 mg treatment, ambulatory SBP was significantly lower with the low-sodium diet compared to the high-sodium diet. Responder rates were also significantly higher with the low sodium diet.
Ferdinand K.C. [50]	2011 USA RCT	ALI/AML/HCTZ 300/10/25; ALI/AML 300/10	412 US minority Stage 2 hypertension	ALI/AML/HCTZ 300/10/25 mg produced greater msSBP reductions and higher responder rates than ALI/AML 300/10 mg therapy in self-identified US minority patients. Both combinations showed similar tolerability
Fogari R. [51]	2011 Italy RCT	ALI 300; AML 10; ALI/AML	120 Mild-to-moderate hypertension	ALI 300 mg and AML 10 mg monotherapies produced similar SBP/DBP reductions. Their combination induced greater BP reductions than both monotherapies and a lower increase in ankle-foot volume than AML monotherapy. PRA was unaffected by AML, and it was reduced by both ALI monotherapy and ALI/AML combination.
Basile J. [52]	2011 USA RCT	ALI/HCTZ 300/25; HCTZ 25 ± AML	451 (≥55 years old) 160 mm Hg ≤ SBP < 200 mm Hg	ALI/HCTZ 300/25 mg therapy provides significantly greater BP reductions and higher control rates than HCTZ 25 mg monotherapy with or without the optional addition of AML in older patients with stage 2 hypertension.
Townsend R.R. [53]	2011 USA RCT	ALI/HCTZ 300/25; AML 10	860 DM 160 mm Hg ≤ SBP < 200 mm Hg	ALI/HCTZ 300/25 mg produced greater msSBP reductions and higher control rates than AML 10 mg. msDBP reductions were similar in both groups. Both treatments were well tolerated, although AE incidence and discontinuation were higher in the AML group.
Brown M.J. [54]	2011 Multicenter RCT	ALI/Placebo; AML/Placebo; ALI/AML	1254 150 mm Hg ≤ SBP < 180 mm Hg	ALI/AML 300/10 mg treatment showed higher SBP reductions than either monotherapy. ALI/AML combination could be recommended for initial treatment for BP ≥ 150 mm Hg.
Sica D. [55]	2011 Multicenter RCT	ALI 150; 300 ± HCTZ	1955 Mild-to-moderate hypertension	Long-term treatment with ALI with or without additional HCTZ is well tolerated and provides effective BP reductions that are sustained over one year.
Ferdinand K.C. [56]	2011 USA RCT	ALI/HCTZ 300/25; AML 10	332 160 mm Hg ≤ SBP < 200 mm Hg	ALI/HCTZ 300/25 mg and AML 10 mg produced similar msSBP/DBP and 24-h ambulatory SBP reductions. Central SBP reductions, measured in a smaller subgroup, were greater in the ALI/HCTZ arm. BP control rates were similar in both groups.
Segura J. [57]	2011 Spain Non RCT	ALI/AML/CHLOR 300/10/50	76 Treatment-resistant hypertension	ALI/AML/CHLOR 300/10/50 mg treatment showed effective BP lowering effects in patients with treatment-resistant hypertension not responding to spironolactone.
Black H.R. [58]	2011 USA RCT	ALI/AML 300/10; AML 10	443 African American 160 mm Hg ≤ SBP < 200 mm Hg	ALI/AML 300/10 mg produced greater msSBP reductions and higher responder rates than AML 10 mg monotherapy.
Krone W. [59]	2011 Multicenter RCT	ALI 300; IRB 300	141 Hypertension and Metabolic syndrome	ALI 300 mg provided significantly greater BP reductions and higher control rates than IRB 300 mg. Both treatments had similar effects on lipid and glucose profiles.
Drummond W. [60]	2011 USA RCT	VAL/HCTZ/ALI 160/25/300; VAL/HCTZ/Placebo	363 DM msDBP ≥ 95 mm Hg inadequately controlled with VAL/HCTZ	BP reductions with ALI 300 mg added to VAL/HCTZ 160/25 mg were numerically greater compared with placebo added to VAL/HCTZ, but not statistically significant.
Schweizer J. [61]	2011 Germany Non RCT	ALI/HCTZ 300/25 ± AML	123 100 mm Hg ≤ DBP < 110 mm Hg inadequately controlled with CAN/HCTZ	Patients inadequately controlled with CAN/HCTZ 32/25 mg achieved clinically and statistically significant BP reductions with the single pill combination of ALI /HCTZ 300/25 mg and the optional addition of AML 5 mg.
Zhu J.R. [62]	2012 Multicenter RCT	ALI 75; 150; 300; RAM 5	1316 Asian Mild-to-moderate hypertension	ALI in doses of 75,150, and 300 mg produced greater BP reductions and higher control rates than RAM 5 mg, but only the differences with ALI 300 mg were statistically significant.
Flack J.M. [63]	2012 Multicenter RCT	ALI/VAL 300/320; VAL 320	451 Stage 2 hypertension	ALI/VAL 300/320 mg significantly reduced msSBP than VAL 320 mg alone. Even greater differences were observed in 24-h ambulatory BP measurements in a small subgroup of 76 patients.
Düsing R. [64]	2012 Multicenter RCT	ALI 300; TEL 80	822 Essential hypertension	ALI 300 mg and TEL 80 mg produced similar mean ambulatory SBP reductions. During a seven-day treatment withdrawal, ALI showed a more sustained BP-lowering effect than TEL.
Axthelm C. [65]	2012 Multicenter Non RCT	ALI/AML 300/10 ± HCTZ	342 Stage 2 hypertension Not controlled by OLM/AML 40/10 mg	ALI/AML 300/10 mg with the optional addition of HCTZ 12.5 mg achieved clinically and statistically significant BP reductions in patients inadequately controlled by OLM/AML 40/10 mg.
Fogari R. [66]	2012 Italy RCT	ALI 300; AML 10; Placebo	170 DM (50–75 years old) Mild-to-moderate hypertension	ALI and AML both, significantly reduced SBP/DBP with no statistical difference between them. Only ALI reduced QT duration and dispersion.
Pfeiffer D. [67]	2012 Multicenter RCT	ALI/AML 300/10; 150/10; AML 10	847 Mild-to-moderate hypertension Not controlled by AML monotherapy	ALI/AML 300/10 and 150/10 mg provided significantly greater msSBP/DBP reductions than AML 10 mg monotherapy. Higher control rates were achieved in the ALI/AML 300/10 mg arm over the AML 10 mg arm.
Villa G. [68]	2012 Multicenter RCT	ALI 75; 150; 300; Placebo	754 (≥65years old) 150 mm Hg ≤ msSBP < 180 mm Hg	ALI 75, 150, and 300 mg provided significantly greater msSBP/DBP reductions compared to placebo. The estimated minimum effective dose was 81.9 mg.
Lacourcière Y. [69]	2012 Multicenter RCT	ALI/AML/HCTZ; ALI/AML; ALI/HCTZ; AML/HCTZ	1191 Moderate-to-severe hypertension	ALI/AML/HCTZ 300/10/25 mg produced statistically superior msSBP/DBP reductions and higher control rates as compared to the ALI/AML, ALI/HCTZ, and AML/HCTZ dual combinations.
Murray A.V. [70]	2012 Multicenter Non RCT	ALI/AML/HCTZ 300/10/25	564 Moderate-to-severe hypertension	ALI/AML/HCTZ 300/10/25 mg provided statistically significant msSBP/DBP reductions, high BP control rates and was well tolerated.
Glorioso N. [71]	2012 Multicenter RCT	ALI/AML 300/10; 300/5; ALI 300	818 Mild-to-moderate hypertension	ALI/AML 300/10 and 300/5 mg provided significantly greater msSBP/DBP reductions and higher BP control rates than ALI 300 mg alone.
Braun-Dullaeus R.C. [72]	2012 Multicenter RCT	ALI/AML 300/10; AML 10	485 Moderate-to-severe hypertension	ALI/AML 300/10 mg produced significantly greater msSBP/DBP reductions and BP control rates than AML 10 mg monotherapy. Both groups had a similar incidence of AEs.
Kanaoka T. [73]	2012 Japan Non RCT	ALI 150→300	21 Nondiabetic Japanese Mild-to-moderate hypertension	ALI 300 mg significantly reduced clinic, ambulatory and central BP measurements. Brachial-ankle pulse wave velocity, a marker of arterial stiffness, also significantly decreased from baseline.
Littlejohn T.W. III [74]	2013 Multicenter RCT	ALI 150; 300; AML 5; 10; ALI/AML; Placebo	1688 95 mm Hg ≤ msDBP < 110 mm Hg	ALI/AML (150 or 300 mg)/ (5 or 10 mg) combinations provided greater BP reductions than either agent alone.
Mazza A [75]	2013 Italy Non RCT	ALI 150–300	106 Resistant hypertension Mild renal dysfunction	ALI 150–300 mg significantly reduced clinic and ambulatory BP measurements, left ventricular mass index and did not affect eGFR.
Bakris G.L. [76]	2013 USA RCT	ALI/VAL 300/320; VAL 320	1143 DM Hypertension Stage 1 or 2 CKD	ALI/VAL 300/320 mg provided additive BP reductions and similar tolerability to VAL 320 mg alone in diabetic patients with early stage CKD.
Yoshitomi Y. [77]	2013 Japan Non RCT	ALI 150 add-on	43 Resistant hypertension	ALI 150 mg added to the existing regimen provided greater BP control rates and was effective as a fourth or fifth line agent.
Andreadis E.A. [78]	2014 Greece RCT	ALI 300; RAM 5± HCTZ or AML	154 Naïve or 6 mo untreated	ALI based therapy produced similar SBP/DBP reductions to RAM based therapy. Both therapies reduced the ambulatory arterial stiffness index.
Imbalzano E. [79]	2015 Italy RCT	ALI add-on; RAM or LOS add-on	126 DM Microalbuminuria Uncontrolled BP	ALI addition to optimal therapy provided higher BP and microalbuminuria reductions compared with the addition of RAM or LOS.
Mizuno H. [80]	2016 Japan RCT	ALI/AML 150–300/5; AML 10	105 Japanese elderly Essential hypertension	ALI/AML 150–300/5 mg and AML 10 mg produced similar reductions in 24-h SBP, and daytime/nighttime SBP. ALI/AML significantly reduced UACR more than high dose AML, but it was significantly less effective in reducing morning BP surge and early morning BP.
Tani S. [81]	2016 Japan Non RCT	ALI add-on	79 Uncontrolled BP	ALI addition to existing regimens provided higher control rates and made it possible to reduce the number of drugs in treatment combinations. No renal function worsening was observed in patients receiving other RAAS inhibitors.

**Table 2:** Summary of studies.

Study	Type	Arms	Participants	Results
AVOID (2008) [97]	Multicenter RCT	ALI/LOS; Placebo/LOS	599 with DM and nephropathy.	ALI significantly reduced UACR compared to placebo. Both arms showed similar BP reductions and AEs.
ALOFT (2008) [98]	Multicenter RCT	ALI 150; Placebo	302 with NYHA class II-IV HF treated with an ACEI or ARB and a beta blocker.	Significant plasma NT-proBNP reductions were observed in the ALI arm compared to placebo.
ALLAY (2009) [99]	Multicenter RCT	ALI 300; LOS 100; ALI/LOS	465 with hypertension, BMI > 25 kg/m^2^, and increased ventricular wall thickness.	In both ALI and LOS arms similar left ventricular mass reductions were observed. Their combination produced similar results with LOS monotherapy.
AVANT GARDE-TIMI 43 (2010) [100]	Multicenter RCT	ALI; VAL; ALI/VAL; Placebo	1101 after acute coronary syndrome without evident HF.	All groups showed similar reductions of NT-proBNP levels. Active therapy groups had a higher incidence of AEs and similar clinical outcomes.
ASPIRE (2011) [101]	Multicenter RCT	ALI; Placebo	820 post-MI, LVEF ≤ 45%, and regional wall motion abnormalities.	No change in left ventricular end-systolic volume was observed when ALI was added to standard treatment compared to placebo.
ALTITUDE (2012) [102]	Multicenter RCT	ACEI or ARB/ALI; ACEI or ARB/Placebo	8562 with DM, and CKD, and/or CVD.	Discontinued due to higher incidence of AEs and non-fatal strokes in the ALI arm.
AQUARIUS (2013) [103]	Multicenter RCT	ALI 300; Placebo	613 with coronary artery disease, prehypertension, and two additional CVD factors.	ALI compared with placebo did not improve or slow the progression of coronary atherosclerosis.
APOLLO (2014) [104]	Multicenter RCT	ALI/HCTZ or AML; ALI/Placebo; HCTZ or AML/Placebo; Placebo/Placebo	1759 elders, SBP ≥ 130 mm Hg and < 160 mm Hg.	Discontinued early. There may be a benefit for substantial CVD reduction with the use of multiple BP-lowering drugs in elder hypertensive individuals.
ASTRONAUT (2013) [105]	Multicenter RCT	ALI 150→300; Placebo	1639 with a LVEF ≤ 40%, [BNP] ≥ 400 pg/mL or [NT-proBNP] ≥ 1600 pg/mL, and fluid overload symptoms.	ALI addition did not reduce cardiovascular death or HF rehospitalization compared to placebo, and had a higher incidence of AEs.
ATMOSPHERE (2016) [106]	Multicenter RCT	ENA 5 or 10; ALI 300; ENA/ALI	7016 with HF and a reduced ejection fraction.	Noninferiority was not proved for ALI when compared to ENA for the outcome of death from a cardiovascular cause or hospitalization for HF.

## Abbreviations

**ACEI**, Angiotensin-Converting-Enzyme Inhibitor; **AE**, Adverse Event; **ALI**, Aliskiren; **AML**, Amlodipine; **Ang**, Angiotensin; **ARB**, Angiotensin II Receptor Blockers; **ATEN**, Atenolol; **BMI**, Body Mass index; **BP**, Blood Pressure; **CAN**, Candesartan; **CCB**, Calcium Channel Blocker; **CHLOR**, Chlortalidone; **CKD**, Chronic Kidney Disease; **CVD**, Cardiovascular Disease; **DBP**, Diastolic Blood Pressure; **DM**, Diabetes Mellitus; **eGFR**, estimated Glomerular Filtration Rate; **ENA**, Enalapril; **HCTZ**, Hydrochlorothiazide; **HF**, Heart Failure; **IRB**, Irbesartan; **LIS**, Lisinopril; **LOS**, Losartan; **LVEF**, Left Ventricular Ejection Fraction; **msDBP**, mean sitting Diastolic Blood Pressure; **msSBP**, mean sitting Systolic Blood Pressure; **OLM**, Olmesartan; **PRA**, Plasma Renin Activity; **PRC**, Plasma Renin Concentration; **RAAS**, Renin–Angiotensin–Aldosterone System; **RAM**, Ramipril; **RCT**, Randomized Control Trial; **SBP**, Systolic Blood Pressure; **TEL**, Telmisartan; **tPA**, tissue Plasminogen Activator; **UACR**, Urine Albumin-to-Creatinine Ratio; **VAL**, Valsartan.

## References

[1] WHO Global Health Observatory (GHO) Data Raised Blood Pressure; Situation and Trends. http://www.who.int/gho/ncd/risk_factors/blood_pressure_prevalence_text/en/.

[2] Lim S.S., Vos T., Flaxman A.D., Danaei G., Shibuya K., Adair-Rohani H., Amann M., Anderson H.R., Andrews K.G., Aryee M. (2012). A comparative risk assessment of burden of disease and injury attributable to 67 risk factors and risk factor clusters in 21 regions, 1990–2010: A systematic analysis for the Global Burden of Disease Study 2010. Lancet.

[3] World Health Organization (2016). World Health Statistics—Monitoring Health For The Sdgs.

[4] Wright J.T., Williamson J.D., Whelton P.K., Snyder J.K., Sink K.M., Rocco M.V., Reboussin D.M., Rahman M., Oparil S., SPRINT Research Group (2015). A Randomized Trial of Intensive versus Standard Blood-Pressure Control. N. Engl. J. Med..

[5] Nguyen G., Delarue F., Burcklé C., Bouzhir L., Giller T., Sraer J.-D. (2002). Pivotal role of the renin/prorenin receptor in angiotensin II production and cellular responses to renin. J. Clin. Investig..

[6] Danser A.H.J. (2015). The Role of the (Pro)renin Receptor in Hypertensive Disease. Am. J. Hypertens..

[7] Verma S., Gupta M., Holmes D.T., Xu L., Teoh H., Gupta S., Yusuf S., Lonn E.M. (2011). Plasma renin activity predicts cardiovascular mortality in the Heart Outcomes Prevention Evaluation (HOPE) study. Eur. Heart J..

[8] Masson S., Solomon S., Angelici L., Latini R., Anand I.S., Prescott M., Maggioni A.P., Tognoni G., Cohn J.N. (2010). The Val-Heft Investigators. Investigators Elevated Plasma Renin Activity Predicts Adverse Outcome in Chronic Heart Failure, Independently of Pharmacologic Therapy: Data From the Valsartan Heart Failure Trial (Val-HeFT). J. Card. Fail..

[9] Bhandari S.K., Batech M., Shi J., Jacobsen S.J., Sim J.J. (2016). Plasma renin activity and risk of cardiovascular and mortality outcomes among individuals with elevated and nonelevated blood pressure. Kidney Res. Clin. Pract..

[10] Hollenberg N.K., Fisher N.D., Price D.A. (1998). Pathways for angiotensin II generation in intact human tissue: Evidence from comparative pharmacological interruption of the renin system. Hypertension.

[11] Rahuel J., Rasetti V., Maibaum J., Rüeger H., Göschke R., Cohen N.C., Stutz S., Cumin F., Fuhrer W., Wood J.M. (2000). Structure-based drug design: The discovery of novel nonpeptide orally active inhibitors of human renin. Chem. Biol..

[12] Wood J.M., Maibaum J., Rahuel J., Grütter M.G., Cohen N.-C., Rasetti V., Rüger H., Göschke R., Stutz S., Fuhrer W. (2003). Structure-based design of aliskiren, a novel orally effective renin inhibitor. Biochem. Biophys. Res. Commun..

[13] Waldmeier F., Glaenzel U., Wirz B., Oberer L., Schmid D., Seiberling M., Valencia J., Riviere G.-J., End P., Vaidyanathan S. (2007). Absorption, Distribution, Metabolism, and Elimination of the Direct Renin Inhibitor Aliskiren in Healthy Volunteers. Drug Metab. Dispos..

[14] Vaidyanathan S., Maboudian M., Warren V., Yeh C.-M., Dieterich H.A., Howard D., Dole W.P. (2008). A study of the pharmacokinetic interactions of the direct renin inhibitor aliskiren with metformin, pioglitazone and fenofibrate in healthy subjects. Curr. Med. Res. Opin..

[15] Vaidyanathan S., Warren V., Yeh C., Bizot M.-N., Dieterich H.A., Dole W.P. (2007). Pharmacokinetics, Safety, and Tolerability of the Oral Renin Inhibitor Aliskiren in Patients With Hepatic Impairment. J. Clin. Pharmacol..

[16] Vaidyanathan S., Bigler H., Yeh C., Bizot M.-N., Dieterich H.A., Howard D., Dole W.P. (2007). Pharmacokinetics of the oral direct renin inhibitor aliskiren alone and in combination with irbesartan in renal impairment. Clin. Pharmacokinet..

[17] Limoges D., Dieterich H.A., Yeh C.-M., Vaidyanathan S., Howard D., Dole W.P. (2008). A study of dose-proportionality in the pharmacokinetics of the oral direct renin inhibitor aliskiren in healthy subjects. Int. J. Clin. Pharmacol. Ther..

[18] Nussberger J., Wuerzner G., Jensen C., Brunner H.R. (2002). Angiotensin II suppression in humans by the orally active renin inhibitor Aliskiren (SPP100): Comparison with enalapril. Hypertension.

[19] Oh B.-H., Mitchell J., Herron J.R., Chung J., Khan M., Keefe D.L. (2007). Aliskiren, an Oral Renin Inhibitor, Provides Dose-Dependent Efficacy and Sustained 24-hour Blood Pressure Control in Patients with Hypertension. J. Am. Coll. Cardiol..

[20] Stanton A.V., Gradman A.H., Schmieder R.E., Nussberger J., Sarangapani R., Prescott M.F. (2010). Aliskiren Monotherapy Does Not Cause Paradoxical Blood Pressure Rises: Meta-Analysis of Data From 8 Clinical Trials. Hypertension.

[21] Danser A.H.J., Charney A., Feldman D.L., Nussberger J., Fisher N., Hollenberg N. (2008). The Renin Rise with Aliskiren: It’s Simply Stoichiometry. Hypertension.

[22] Stanton A., Jensen C., Nussberger J., O’Brien E. (2003). Blood Pressure Lowering in Essential Hypertension with an Oral Renin Inhibitor, Aliskiren. Hypertension.

[23] Gradman A.H., Schmieder R.E., Lins R.L., Nussberger J., Chiang Y., Bedigian M.P. (2005). Aliskiren, a Novel Orally Effective Renin Inhibitor, Provides Dose-Dependent Antihypertensive Efficacy and Placebo-Like Tolerability in Hypertensive Patients. Circulation.

[24] Kushiro T., Itakura H., Abo Y., Gotou H., Terao S., Keefe D.L. (2006). Aliskiren, a Novel Oral Renin Inhibitor, Provides Dose-Dependent Efficacy and Placebo-Like Tolerability in Japanese Patients with Hypertension. Hypertens. Res..

[25] Pool J., Schmieder R., Azizi M., Aldigier J., Januszewicz A., Zidek W., Chiang Y., Satlin A. (2007). Aliskiren, an Orally Effective Renin Inhibitor, Provides Antihypertensive Efficacy Alone and in Combination With Valsartan. Am. J. Hypertens..

[26] O’Brien E., Barton J., Nussberger J., Mulcahy D., Jensen C., Dicker P., Stanton A. (2007). Aliskiren Reduces Blood Pressure and Suppresses Plasma Renin Activity in Combination With a Thiazide Diuretic, an Angiotensin-Converting Enzyme Inhibitor, or an Angiotensin Receptor Blocker. Hypertension.

[27] Jordan J., Engeli S., Boye S.W., le Breton S., Keefe D.L. (2007). Direct Renin Inhibition With Aliskiren in Obese Patients With Arterial Hypertension. Hypertension.

[28] Strasser R.H., Puig J.G., Farsang C., Croket M., Li J., van Ingen H. (2007). A comparison of the tolerability of the direct renin inhibitor aliskiren and lisinopril in patients with severe hypertension. J. Hum. Hypertens..

[29] Verdecchia P., Calvo C., Mockel V., Keeling L., Satlin A. (2007). Safety and efficacy of the oral direct renin inhibitor aliskiren in elderly patients with hypertension. Blood Press..

[30] Drummond W., Munger M.A., Rafique Essop M., Maboudian M., Khan M., Keefe D.L. (2007). Antihypertensive efficacy of the oral direct renin inhibitor aliskiren as add-on therapy in patients not responding to amlodipine monotherapy. J. Clin. Hypertens..

[31] Oparil S., Yarows S.A., Patel S., Fang H., Zhang J., Satlin A. (2007). Efficacy and safety of combined use of aliskiren and valsartan in patients with hypertension: A randomised, double-blind trial. Lancet.

[32] Uresin Y., Taylor A., Kilo C., Tschöpe D., Santonastaso M., Ibram G., Fang H., Satlin A. (2007). Efficacy and safety of the direct renin inhibitor aliskiren and ramipril alone or in combination in patients with diabetes and hypertension. J. Renin-Angiotensin-Aldosterone Syst..

[33] Villamil A., Chrysant S.G., Calhoun D., Schober B., Hsu H., Matrisciano-Dimichino L., Zhang J. (2007). Renin inhibition with aliskiren provides additive antihypertensive efficacy when used in combination with hydrochlorothiazide. J. Hypertens..

[34] Andersen K., Weinberger M.H., Egan B., Constance C.M., Ali M.A., Jin J., Keefe D.L. (2008). Comparative efficacy and safety of aliskiren, an oral direct renin inhibitor, and ramipril in hypertension: A 6-month, randomized, double-blind trial. J. Hypertens..

[35] Dietz R., Dechend R., Yu C.-M., Bheda M., Ford J., Prescott M.F., Keefe D.L. (2008). Effects of the direct renin inhibitor aliskiren and atenolol alone or in combination in patients with hypertension1. J. Renin-Angiotensin-Aldosterone Syst..

[36] Nickenig G., Simanenkov V., Lembo G., Rodriguez P., Salko T., Ritter S., Zhang J. (2008). Efficacy of aliskiren/hydrochlorothiazide single-pill combinations in aliskiren non-responders. Blood Press. Suppl..

[37] Geiger H., Barranco E., Gorostidi M., Taylor A., Zhang X., Xiang Z., Zhang J. (2009). Combination Therapy With Various Combinations of Aliskiren, Valsartan, and Hydrochlorothiazide in Hypertensive Patients Not Adequately Responsive to Hydrochlorothiazide Alone. J. Clin. Hypertens..

[38] Kushiro T., Itakura H., Abo Y., Gotou H., Terao S., Keefe D.L. (2009). Long-term safety, tolerability, and antihypertensive efficacy of aliskiren, an oral direct renin inhibitor, in Japanese patients with hypertension. Hypertens. Res..

[39] Puig J.G., Schunkert H., Taylor A.A., Boye S., Jin J., Keefe D.L. (2009). Evaluation of the dose—Response relationship of aliskiren, a direct renin inhibitor, in an 8-week, multicenter, randomized, double-blind, parallel-group, placebo-controlled study in adult patients with stage 1 or 2 essential hypertension. Clin. Ther..

[40] Blumenstein M., Romaszko J., Calderón A., Andersen K., Ibram G., Liu Z., Zhang J. (2009). Antihypertensive efficacy and tolerability of aliskiren/hydrochlorothiazide (HCT) single-pill combinations in patients who are non-responsive to HCT 25 mg alone. Curr. Med. Res. Opin..

[41] Littlejohn T.W., Trenkwalder P., Hollanders G., Zhao Y., Liao W. (2009). Long-term safety, tolerability and efficacy of combination therapy with aliskiren and amlodipine in patients with hypertension. Curr. Med. Res. Opin..

[42] Schmieder R.E., Philipp T., Guerediaga J., Gorostidi M., Smith B., Weissbach N., Maboudian M., Botha J., van Ingen H. (2009). Long-Term Antihypertensive Efficacy and Safety of the Oral Direct Renin Inhibitor Aliskiren: A 12-Month Randomized, Double-Blind Comparator Trial With Hydrochlorothiazide. Circulation.

[43] Duprez D.A., Munger M.A., Botha J., Keefe D.L., Charney A.N. (2010). Aliskiren for Geriatric Lowering of Systolic Hypertension: A randomized controlled trial. J. Hum. Hypertens..

[44] Black H.R., Kribben A., Aguirre Palacios F., Bijarnia M., Laflamme A.K., Baschiera F. (2010). Aliskiren Alone or in Combination With Hydrochlorothiazide in Patients With the Lower Ranges of Stage 2 Hypertension: The ACQUIRE Randomized Double-Blind Study. J. Clin. Hypertens..

[45] Ito S., Nakura N., Le Breton S., Keefe D. (2010). Efficacy and safety of aliskiren in Japanese hypertensive patients with renal dysfunction. Hypertens. Res..

[46] Chrysant S.G., Murray A.V., Hoppe U.C., Dattani D., Patel S., Ritter S., Zhang J. (2010). Long-term safety and efficacy of aliskiren and valsartan combination with or without the addition of HCT in patients with hypertension. Curr. Med. Res. Opin..

[47] Palatini P., Jung W., Shlyakhto E., Botha J., Bush C., Keefe D.L. (2010). Maintenance of blood-pressure-lowering effect following a missed dose of aliskiren, irbesartan or ramipril: Results of a randomized, double-blind study. J. Hum. Hypertens..

[48] Fogari R., Zoppi A., Mugellini A., Lazzari P., Derosa G. (2010). Different Effects of Aliskiren and Losartan on Fibrinolysis and Insulin Sensitivity in Hypertensive Patients with Metabolic Syndrome. Horm. Metab. Res..

[49] Weir M.R., Yadao A.M., Purkayastha D., Charney A.N. (2010). Effects of High- and Low-Sodium Diets on Ambulatory Blood Pressure in Patients With Hypertension Receiving Aliskiren. J. Cardiovasc. Pharmacol. Ther..

[50] Ferdinand K.C., Weitzman R., Israel M., Lee J., Purkayastha D., Jaimes E.A. (2011). Efficacy and safety of aliskiren-based dual and triple combination therapies in US minority patients with stage 2 hypertension. J. Am. Soc. Hypertens..

[51] Fogari R., Zoppi A., Mugellini A., Maffioli P., Lazzari P., Monti C., Derosa G. (2011). Effect of aliskiren addition to amlodipine on ankle edema in hypertensive patients: A three-way crossover study. Expert Opin. Pharmacother..

[52] Basile J., Babazadeh S., Lillestol M., Botha J., Yurkovic C., Weitzman R. (2011). Comparison of Aliskiren/Hydrochlorothiazide Combination Therapy With Hydrochlorothiazide Monotherapy in Older Patients With Stage 2 Systolic Hypertension: Results of the ACTION Study. J. Clin. Hypertens..

[53] Townsend R.R., Forker A.D., Bhosekar V., Yadao A., Keefe D.L. (2011). Comparison of Aliskiren/Hydrochlorothiazide Combination Therapy and Amlodipine Monotherapy in Patients With Stage 2 Systolic Hypertension and Type 2 Diabetes Mellitus. J. Clin. Hypertens..

[54] Brown M.J., McInnes G.T., Papst C.C., Zhang J., MacDonald T.M. (2011). Aliskiren and the calcium channel blocker amlodipine combination as an initial treatment strategy for hypertension control (ACCELERATE): A randomised, parallel-group trial. Lancet.

[55] Sica D., Gradman A.H., Lederballe O., Kolloch R.E., Zhang J., Keefe D.L. (2011). Long-Term Safety and Tolerability of the Oral Direct Renin Inhibitor Aliskiren with Optional Add-On Hydrochlorothiazide in Patients with Hypertension. Clin. Drug Investig..

[56] Ferdinand K.C., Pool J., Weitzman R., Purkayastha D., Townsend R. (2011). Peripheral and Central Blood Pressure Responses of Combination Aliskiren/Hydrochlorothiazide and Amlodipine Monotherapy in African American Patients With Stage 2 Hypertension: The ATLAAST Trial. J. Clin. Hypertens..

[57] Segura J., Cerezo C., Garcia-Donaire J.A., Schmieder R.E., Praga M., de la Sierra A., Ruilope L.M. (2011). Validation of a therapeutic scheme for the treatment of resistant hypertension. J. Am. Soc. Hypertens..

[58] Black H.R., Weinberger M.H., Purkayastha D., Lee J., Sridharan K., Israel M., Hilkert R., Izzo J. (2011). Comparative Efficacy and Safety of Combination Aliskiren/Amlodipine and Amlodipine Monotherapy in African Americans With Stage 2 Hypertension. J. Clin. Hypertens..

[59] Krone W., Hanefeld M., Meyer H.-F., Jung T., Bartlett M., Yeh C.-M., Rajman I., Prescott M.F., Dole W.P. (2011). Comparative efficacy and safety of aliskiren and irbesartan in patients with hypertension and metabolic syndrome. J. Hum. Hypertens..

[60] Drummond W., Sirenko Y.M., Ramos E., Baek I., Keefe D.L. (2011). Aliskiren as Add-On Therapy in the Treatment of Hypertensive Diabetic Patients Inadequately Controlled with Valsartan/HCT Combination. Am. J. Cardiovasc. Drugs.

[61] Schweizer J., Ulmer H.-J., Benduhn H., Klebs S. (2011). Efficacy and tolerability of aliskiren 300 mg/hydrochlorothiazide 25 mg (±amlodipine 5 mg) in hypertensive patients not controlled by candesartan 32 mg plus HCT 25 mg. Curr. Med. Res. Opin..

[62] Zhu J.-R., Sun N.-L., Yang K., Hu J., Xu G., Hong H., Wang R., Tu Y.-M., Ritter S., Keefe D. (2012). Efficacy and safety of aliskiren, a direct renin inhibitor, compared with ramipril in Asian patients with mild to moderate hypertension. Hypertens. Res..

[63] Flack J.M., Yadao A.M., Purkayastha D., Samuel R., White W.B. (2012). Comparison of the effects of aliskiren/valsartan in combination versus valsartan alone in patients with Stage 2 hypertension. J. Am. Soc. Hypertens..

[64] Düsing R., Brunel P., Baek I., Baschiera F. (2012). Sustained decrease in blood pressure following missed doses of aliskiren or telmisartan. J. Hypertens..

[65] Axthelm C., Sieder C., Meister F., Kaiser E. (2012). Efficacy and tolerability of the single-pill combination of aliskiren 300 mg/amlodipine 10 mg in hypertensive patients not controlled by olmesartan 40 mg/amlodipine 10 mg. Curr. Med. Res. Opin..

[66] Fogari R., Zoppi A., Maffioli P., Monti C., Lazzari P., Mugellini A., Derosa G. (2012). Effects of aliskiren on QT duration and dispersion in hypertensive patients with type 2 diabetes mellitus. Diabetes Obes. Metab..

[67] Pfeiffer D., Rennie N., Papst C.C., Zhang J. (2012). Efficacy and tolerability of aliskiren/amlodipine single-pill combinations in patients who did not respond fully to amlodipine monotherapy¥. Curr. Vasc. Pharmacol..

[68] Villa G., Breton S., Le Ibram G., Keefe D.L. (2012). Efficacy, Safety, and Tolerability of Aliskiren Monotherapy Administered With a Light Meal in Elderly Hypertensive Patients: A Randomized, Double-Blind, Placebo-Controlled, Dose-Response Evaluation Study. J. Clin. Pharmacol..

[69] Lacourcière Y., Taddei S., Konis G., Fang H., Severin T., Zhang J. (2012). Clinic and ambulatory blood pressure lowering effect of aliskiren/amlodipine/hydrochlorothiazide combination in patients with moderate-to-severe hypertension. J. Hypertens..

[70] Murray A.V., Koenig W., Garcia-Puig J., Patel S., Zhang J. (2012). Safety and Efficacy of Aliskiren/Amlodipine/Hydrochlorothiazide Triple Combination in Patients With Moderate to Severe Hypertension: A 54-Week, Open-Label Study. J. Clin. Hypertens..

[71] Glorioso N., Thomas M., Troffa C., Argiolas G., Patel S., Baek I., Zhang J. (2012). Antihypertensive efficacy and tolerability of aliskiren/amlodipine single-pill combinations in patients with an inadequate response to aliskiren monotherapy. Curr. Vasc. Pharmacol..

[72] Braun-Dullaeus R.C., Shustov S.B., Alvarez C., Rogelio G.G., Zhang J., Hristoskova S., Häring D.A. (2012). Treatment with aliskiren/amlodipine combination in patients with moderate-to-severe hypertension: A randomised, double-blind, active comparator trial. Int. J. Clin. Pract..

[73] Kanaoka T., Tamura K., Ohsawa M., Wakui H., Maeda A., Dejima T., Azushima K., Haku S., Mitsuhashi H., Yanagi M. (2012). Effects of Aliskiren-Based Therapy on Ambulatory Blood Pressure Profile, Central Hemodynamics, and Arterial Stiffness in Nondiabetic Mild to Moderate Hypertensive Patients. J. Clin. Hypertens..

[74] Littlejohn T.W., Jones S.W., Zhang J., Hsu H., Keefe D.L. (2013). Efficacy and safety of aliskiren and amlodipine combination therapy in patients with hypertension: A randomized, double-blind, multifactorial study. J. Hum. Hypertens..

[75] Mazza A., Montemurro D., Zuin M., Schiavon L., Zorzan S., Chondrogiannis S., Ferretti A., Ramazzina E., Rubello D. (2013). Aliskiren improves blood pressure control and prevents cardiac damage in high-risk hypertensive subjects. Minerva Cardioangiol..

[76] Bakris G.L., Oparil S., Purkayastha D., Yadao A.M., Alessi T., Sowers J.R. (2013). Randomized Study of Antihypertensive Efficacy and Safety of Combination Aliskiren/Valsartan vs Valsartan Monotherapy in Hypertensive Participants With Type 2 Diabetes Mellitus. J. Clin. Hypertens..

[77] Yoshitomi Y., Kawanishi K., Yamaguchi A., Sakurai S., Minai K., Ishii T., Tarutani Y., Tsujibayashi T., Kaneki M., Saitou Y. (2013). Effectiveness of the direct renin inhibitor, aliskiren, in patients with resistant hypertension. Int. Heart J..

[78] Andreadis E.A., Angelopoulos E.T., Kolyvas G.N., Agaliotis G.D., Mousoulis C.G., Mousoulis G.P. (2014). The effect of aliskiren versus ramipril-based treatment on the Ambulatory Arterial Stiffness Index in hypertensive patients. Int. Angiol..

[79] Imbalzano E., Scarpelli M., Mandraffino G., Creazzo M., Lizio G., Trapani G., Dattilo G., Dalbeni A., Tomasello C., Sardo M.A. (2015). Combination therapy with aliskiren versus ramipril or losartan added to conventional therapy in patients with type 2 diabetes mellitus, uncontrolled hypertension and microalbuminuria. J. Renin-Angiotensin-Aldosterone Syst..

[80] Mizuno H., Hoshide S., Fukutomi M., Kario K. (2016). Differing Effects of Aliskiren/Amlodipine Combination and High-Dose Amlodipine Monotherapy on Ambulatory Blood Pressure and Target Organ Protection. J. Clin. Hypertens..

[81] Tani S., Kushiro T., Takahashi A., Kawamata H., Ohkubo K., Nagao K., Hirayama A. (2016). Antihypertensive Efficacy of the Direct Renin Inhibitor Aliskiren as Add-on Therapy in Patients with Poorly Controlled Hypertension. Intern. Med..

[82] Musini V.M., Fortin P.M., Bassett K., Wright J.M. (2009). Blood pressure lowering efficacy of renin inhibitors for primary hypertension: A Cochrane systematic review. J. Hum. Hypertens..

[83] Verdecchia P., Angeli F., Mazzotta G., Martire P., Garofoli M., Gentile G., Reboldi G. (2010). Aliskiren versus ramipril in hypertension. Ther. Adv. Cardiovasc. Dis..

[84] Gao D., Ning N., Niu X., Wei J., Sun P., Hao G. (2011). Aliskiren vs. Angiotensin Receptor Blockers in Hypertension: Meta-Analysis of Randomized Controlled Trials. Am. J. Hypertens..

[85] Zheng Z., Shi H., Jia J., Li D., Lin S. (2011). A systematic review and meta-analysis of aliskiren and angiotension receptor blockers in the management of essential hypertension. J. Renin-Angiotensin-Aldosterone Syst..

[86] Chen Y., Meng L., Shao H., Yu F. (2013). Aliskiren vs. other antihypertensive drugs in the treatment of hypertension: A meta-analysis. Hypertens. Res..

[87] Liu Y., Chen K., Kou X., Han Y., Zhou L., Zeng C. (2013). Aliskiren and Amlodipine in the Management of Essential Hypertension: Meta-Analysis of Randomized Controlled Trials. PLoS ONE.

[88] Liu Y., Yan R., Song A., Niu X., Cao C., Wei J., Dong X., Gao D. (2014). Aliskiren/Amlodipine vs. Aliskiren/Hydrochlorothiazide in Hypertension: Indirect Meta-Analysis of Trials Comparing the Two Combinations vs. Monotherapy. Am. J. Hypertens..

[89] Makani H., Bangalore S., Desouza K.A., Shah A., Messerli F.H. (2013). Efficacy and safety of dual blockade of the renin-angiotensin system: Meta-analysis of randomised trials. BMJ.

[90] Bramlage P., Pittrow D., Wittchen H., Kirch W., Boehler S., Lehnert H., Hoefler M., Unger T., Sharma A. (2004). Hypertension in overweight and obese primary care patients is highly prevalent and poorly controlled. Am. J. Hypertens..

[91] Schmieder R.E., Philipp T., Guerediaga J., Gorostidi M., Bush C., Keefe D.L. (2009). Aliskiren-based therapy lowers blood pressure more effectively than hydrochlorothiazide-based therapy in obese patients with hypertension: Sub-analysis of a 52-week, randomized, double-blind trial. J. Hypertens..

[92] Verpooten G.A., Aerts A., Coen N., Vancayzeele S., Hermans C., Bowles J., MacDonald K., Abraham I., Lee C.S. (2011). Antihypertensive effectiveness of aliskiren for the “real-world” management of hypertension: Multilevel modelling of 180-day blood pressure outcomes (the Belgian DRIVER Study). Int. J. Clin. Pract..

[93] Volpe M., Tocci G., Bianchini F., De Rosa M., Fedozzi E., Covezzoli A., Maggioni A.P. (2011). AIFA Drug Monitoring Program: Aliskiren Registry Use of aliskiren in a “real-life” model of hypertension management. J. Hypertens..

[94] Maddury S.R., Pande A., Haque K.M., Echtay A., Go L., Gulzar T., Kadwa M., Hristoskova S. (2013). Effectiveness and Safety of Aliskiren and Aliskiren Hydrochlorothiazide (HCT) in a Multiethnic, Real-World Setting. Adv. Ther..

[95] Zeymer U., Dechend R., Riemer T., Kaiser E., Senges J., Pittrow D., Schmieder R.E. (2014). 3A Registry Investigators 1-Year outcomes of hypertension management in 13,000 outpatients under practice conditions: Prospective 3A registry. Int. J. Cardiol..

[96] Rosenkranz A.R., Ratzinger M. (2015). Efficacy, safety, and tolerability of antihypertensive therapy with aliskiren/amlodipine in clinical practice in Austria. The RALLY (Rasilamlo long lasting efficacy) study. Wien. Klin. Wochenschr..

[97] Parving H.-H., Persson F., Lewis J.B., Lewis E.J., Hollenberg N.K. (2008). AVOID Study Investigators Aliskiren Combined with Losartan in Type 2 Diabetes and Nephropathy. N. Engl. J. Med..

[98] McMurray J.J.V., Pitt B., Latini R., Maggioni A.P., Solomon S.D., Keefe D.L., Ford J., Verma A., Lewsey J. (2008). Aliskiren Observation of Heart Failure Treatment (ALOFT) Investigators Effects of the Oral Direct Renin Inhibitor Aliskiren in Patients With Symptomatic Heart Failure. Circ. Hear. Fail..

[99] Solomon S.D., Appelbaum E., Manning W.J., Verma A., Berglund T., Lukashevich V., Cherif Papst C., Smith B.A., Dahlof B. (2009). Aliskiren in Left Ventricular Hypertrophy (ALLAY) Trial Investigators Effect of the Direct Renin Inhibitor Aliskiren, the Angiotensin Receptor Blocker Losartan, or Both on Left Ventricular Mass in Patients With Hypertension and Left Ventricular Hypertrophy. Circulation.

[100] Scirica B.M., Morrow D.A., Bode C., Ruzyllo W., Ruda M., Oude Ophuis A.J.M., Lopez-Sendon J., Swedberg K., Ogorek M., Rifai N. (2010). Patients with acute coronary syndromes and elevated levels of natriuretic peptides: The results of the AVANT GARDE-TIMI 43 Trial. Eur. Heart J..

[101] Solomon S.D., Hee S., Shah A., Skali H., Desai A., Kober L., Maggioni A.P., Rouleau J.L., Kelly R.Y., Hester A. (2011). Aliskiren Study in Post-MI Patients to Reduce Remodeling (ASPIRE) Investigators Effect of the direct renin inhibitor aliskiren on left ventricular remodelling following myocardial infarction with systolic dysfunction. Eur. Heart J..

[102] Parving H.-H., Brenner B.M., McMurray J.J.V., de Zeeuw D., Haffner S.M., Solomon S.D., Chaturvedi N., Persson F., Desai A.S., Nicolaides M. (2012). ALTITUDE Investigators Cardiorenal End Points in a Trial of Aliskiren for Type 2 Diabetes. N. Engl. J. Med..

[103] Nicholls S.J., Bakris G.L., Kastelein J.J.P., Menon V., Williams B., Armbrecht J., Brunel P., Nicolaides M., Hsu A., Hu B. (2013). Effect of Aliskiren on Progression of Coronary Disease in Patients With Prehypertension. JAMA.

[104] Teo K.K., Pfeffer M., Mancia G., O’Donnell M., Dagenais G., Diaz R., Dans A., Liu L., Bosch J., Joseph P. (2014). Aliskiren Prevention of Later Life Outcomes trial Investigators Aliskiren alone or with other antihypertensives in the elderly with borderline and stage 1 hypertension: The APOLLO trial. Eur. Heart J..

[105] Gheorghiade M., Böhm M., Greene S.J., Fonarow G.C., Lewis E.F., Zannad F., Solomon S.D., Baschiera F., Botha J., Hua T.A. (2013). ASTRONAUT Investigators and Coordinators, for the Effect of Aliskiren on Postdischarge Mortality and Heart Failure Readmissions Among Patients Hospitalized for Heart Failure. JAMA.

[106] McMurray J.J.V., Krum H., Abraham W.T., Dickstein K., Køber L.V., Desai A.S., Solomon S.D., Greenlaw N., Ali M.A., Chiang Y. (2016). ATMOSPHERE Committees Investigators Aliskiren, Enalapril, or Aliskiren and Enalapril in Heart Failure. N. Engl. J. Med..

[107] McMurray J.J.V., Abraham W.T., Dickstein K., Køber L., Massie B.M., Krum H. (2012). Aliskiren, ALTITUDE, and the implications for ATMOSPHERE. Eur. J. Heart Fail..

[108] Raptis A.E., Markakis K.P., Mazioti M.C., Ikonomidis I., Maratou E.P., Vlahakos D.V., Kotsifaki E.E., Voumvourakis A.N., Tsirogianni A.G., Lambadiari V.A. (2015). Effect of Aliskiren on Circulating Endothelial Progenitor Cells and Vascular Function in Patients With Type 2 Diabetes and Essential Hypertension. Am. J. Hypertens..

[109] Bonadei I., Vizzardi E., D’Aloia A., Sciatti E., Raddino R., Metra M. (2014). Role of Aliskiren on Arterial Stiffness and Endothelial Function in Patients With Primary Hypertension. J. Clin. Hypertens..

[110] Fukutomi M., Hoshide S., Mizuno H., Kario K. (2014). Differential Effects of Aliskiren/Amlodipine Combination and High-Dose Amlodipine Monotherapy on Endothelial Function in Elderly Hypertensive Patients. Am. J. Hypertens..

